# Quantitative phage-host interaction profiling supports the design of a broadly active phage cocktail against multidrug-resistant *Enterococcus faecalis* from poultry

**DOI:** 10.3389/fmicb.2026.1924276

**Published:** 2026-09-21

**Authors:** Josep Garcia-Llorens, Ignacio Samuel Gómez-Cano, Jan Torres-Boncompte, Jose M. Soriano, Pablo Catalá-Gregori, Sandra Sevilla-Navarro

**Affiliations:** 1 Centro de Calidad Avícola y Alimentación Animal de la Comunidad Valenciana, (CECAV), Castellón, Spain; 2 Food & Health Lab, Institute of Materials Science, University of Valencia, Valencia, Spain; 3 Joint Research Unit on Endocrinology, Nutrition and Clinical Dietetics, University of Valencia-Health Research Institute La Fe, Valencia, Spain; 4 Departamento de Producción y Sanidad Animal, Salud Pública Veterinaria y Ciencia y Tecnología de los Alimentos, Instituto de Ciencias Biomédicas, Facultad de Veterinaria, Universidad Cardenal Herrera-CEU, CEU Universities, Moncada, Spain

**Keywords:** antimicrobial resistance, *Enterococcus*, genomic analysis, host range, phage therapy, poultry production

## Abstract

**Introduction:**

*Enterococcus faecalis* is an opportunistic pathogen of One Health relevance and an important reservoir of antimicrobial resistance in poultry production systems. In this study, we generated a large-scale quantitative phage-host interaction dataset to support the rational design of phage cocktails targeting multidrug-resistant poultry-associated *E. faecalis*.

**Methods:**

Thirty-eight lytic phages were isolated from poultry farm samples, genomically characterized, and evaluated against 51 clinical poultry isolates using efficiency of plating as a quantitative measure of productive infectivity.

**Results:**

The complete phage collection achieved 94.12% cumulative strain coverage, while genomic relatedness showed only a moderate association with infectivity profiles, highlighting the need for functional screening. Computational optimization identified a six-phage cocktail, CKT6, which displayed broad *in vitro* activity against diverse strain backgrounds, reaching 92.16% coverage at MOI 1. Importantly, cocktail performance was not fully predictable from individual phage activity, as host-dependent synergistic and antagonistic effects were observed.

**Discussion:**

These findings support quantitative phage-host interaction profiling as a robust framework for reproducible and adaptive phage-cocktail design. Further validation under biofilm-associated, formulation-relevant and field-like poultry-production conditions will be required before practical application.

## 1 Introduction

*Enterococcus* spp. are widely distributed in the environment and are recognized as important opportunistic pathogens in both humans and animals (Byappanahalli et al., 2012). Among them, *Enterococcus faecalis* (*E. faecalis*) stands out for its ability to express multiple virulence factors, including biofilm formation and cytolysin production, which promote colonization, persistence, and tissue damage (Reynolds et al., 2025). In poultry, this pathogenic potential is well documented, as *E. faecalis* is associated with a wide range of disease conditions, including septicaemia, endocarditis, arthritis, and spondylitis, leading to significant economic losses due to increased mortality and reduced productivity across different production systems (Stȩpień-Pyśniak et al., 2016; Nielsen et al., 2022; Bzdil et al., 2023).

Beyond poultry production, *E. faecalis* is also of considerable clinical relevance in humans. Human enterococcal infections commonly include urinary tract infections, bacteraemia, infective endocarditis, and wound or intra-abdominal infections, and their treatment is often complicated by intrinsic resistance to several antimicrobial classes as well as the ability to acquire and disseminate additional resistance determinants [Centers for Disease Control and Prevention (CDC), 2024; Said et al., 2024]. From a One Health perspective, this connection between human and poultry-associated *E. faecalis* is particularly relevant, as enterococci are frequently detected in poultry meat, and chicken-associated isolates may display multidrug resistance, virulence traits, and genetic relatedness to lineages of clinical concern (Bortolaia et al., 2016). Controlling *E. faecalis* at the poultry-production level may have implications beyond animal health, contributing to the reduction of potential reservoirs of resistant enterococci and resistance genes along the food chain (Poulsen et al., 2012).

The rising prevalence of antimicrobial resistance (AMR) in *E. faecalis* represents a major challenge in poultry production, as it compromises both disease control and treatment effectiveness (Jung et al., 2018). The rise of resistance significantly reduces available therapeutic options and is associated with increased morbidity and mortality (Dupont et al., 2011; Garcia-Llorens et al., 2025). Moreover, treatment outcomes are often suboptimal, particularly when infections are not detected early, and are further hindered by the ability of *E. faecalis* to persist in biofilms and farm environments (Bzdil et al., 2023; Morishita, 2025; Reynolds et al., 2025). These factors not only impact animal health and productivity but also highlight the broader implications of AMR, including the potential risk of zoonotic transmission (Martinez-Laorden et al., 2023; Kerek et al., 2024). In this context, the limitations of conventional antimicrobials, combined with the lack of effective vaccines and broadly implemented prophylactic strategies, underscore the urgent need for alternative control strategies (Nielsen et al., 2022).

Bacteriophages, viruses that specifically infect bacteria, have emerged as promising biological tools for the control of multidrug-resistant pathogens (Lavilla et al., 2023). Their key advantages include high host specificity, the capacity to self-amplify at the site of infection, and a favourable safety profile (Kutter et al., 2010; Uyttebroek et al., 2022; Rodríguez-Lucas and Ladero, 2023), making them particularly attractive for application in complex environments such as poultry production systems. Accordingly, bacteriophages have gained considerable attention as a promising strategy for controlling *E. faecalis* in poultry systems and derived products (Rodríguez-Lucas and Ladero, 2023; Li et al., 2026). Despite the increasing interest in phage-based control strategies, the design of effective phage cocktails remains challenging due to the marked field-level diversity of *E. faecalis* and the limited predictability of phage host range. In particular, for poultry-associated *E. faecalis*, there is a lack of systematically generated, efficiency of plating (EOP)-based quantitative infectivity datasets across large and diverse clinical strain panels that could inform rational, computationally guided cocktail selection.

Thus, the present study addressed this gap by generating a large collection of phages and evaluating their activity against a diverse panel of clinical poultry isolates. To this end, quantitative infectivity data, genomic analyses, and computational optimization approaches were integrated to identify a broadly active phage cocktail against multidrug-resistant *E. faecalis*. Furthermore, phage-host interaction patterns were evaluated to determine their utility for the rational design of robust phage cocktails.

## 2 Materials and methods

### 2.1 Bacterial strains and culture conditions

A total of 51 clinical *E. faecalis* strains were included in this study. These isolates originated from the collection previously described by Garcia-Llorens et al. (2025), which comprised strains recovered from different poultry farms across Spain, providing broad national representation. Strains were selected based on production system (layers, breeders, and broilers), virulence profiles, and multidrug resistance characteristics.

All strains were reactivated by triple streaking on Columbia Blood Agar (CA) (Oxoid Deutschland GmbH, Wesel, Germany) plates and incubated overnight (ON) at 37.5 °C. The resulting cultures were subsequently stored at 4 °C for further analyses.

### 2.2 Source of phage isolation

Phages were isolated from poultry samples (including faeces, cloacal swabs, drag swabs and litter) collected from commercial poultry farms over a one-year period (2024) as part of the laboratory’s routine diagnostic workflow.

Environmental samples were diluted 1:10 (w/v) in buffered peptone water (BPW, VWR Chemicals, Barcelona, Spain) and incubated ON at 37 °C. Following incubation, 1 mL of each sample was pooled in groups of 10 samples, used as starting material for phage isolation.

### 2.3 Phage isolation, purification and propagation

Samples were centrifuged 8,000 × *g* for 5 min and the supernatant was subsequently filtered through a 0.22 μm pore size polyether sulfone (PES) membrane filter (VWR Chemicals, Barcelona, Spain) to remove any remaining bacteria. To evaluate the lytic potential of the filtered samples, a spot test assay was employed against the *E. faecalis* strains (Mirzaei and Nilsson, 2015). Samples demonstrating clear lytic activity were selected for phage isolation procedures. Each lysate was serially diluted 1:10 in LB broth (Scharlau, Barcelona, Spain) and mixed with 200 μL of the exponential-phase of host bacterial suspension at an optical density at 600 nm (OD_600_) of 0.4 (∼10^8^ CFU/mL). Following an incubation of 15 min period at room temperature to allow phage adsorption, the mixture was incorporated into 5 mL of LB soft agar (0.6% agar; Scharlau, Barcelona, Spain) and overlaid onto LB agar (Scharlau, Barcelona, Spain) plates using the double-layer agar (DLA) (Mirzaei and Nilsson, 2015). Plates were incubated at 37 °C, ON. This purification cycle was repeated three times, until a single purified phage isolate was obtained. Whenever a phage was isolated against a particular strain, it was subsequently evaluated by spot assay against the remaining strains. This process was repeated until a phage collection with coverage of all 51 strains was established.

### 2.4 Host range (HR) and efficiency of plating (EOP)

Host range (HR) was first assessed as a qualitative screening to identify phage-strain combinations showing lytic activity against the propagation strains and the complete panel of challenge *E. faecalis* strains (*n* = 51). Lytic reactions were recorded according to plaque/lysis appearance, and only combinations showing clear lysis (C) were further evaluated by efficiency of plating (EOP), in order to focus on productive infections and avoid overestimating host range based on non-productive lysis or growth inhibition (Kutter, 2009). For EOP determination, serial 1:10 dilutions of the purified phages were carried out, and each dilution was tested through the DLA method. After an ON incubation at 37 °C, both lytic activity and phage titers capabilities were determined. EOP towards the strains was calculated relative to titers the phage reached with its own propagation strains, only when clear and individual plaques were observed (Torres-Boncompte et al., 2025).

### 2.5 Rational design of phage cocktails

Phage-cocktail formulation was based on the experimental assessment of phage lytic activity determined by EOP. EOP values obtained against the tested bacterial strains were classified according to lytic efficiency as follows: values ≥0.5 were considered indicative of high lytic efficiency; values between 0.2 and <0.5 as moderate lytic activity; values between 0.001 and <0.2 as low lytic efficiency; and values <0.001 as non-lytic. Accordingly, EOP values below 0.5 were regarded as suboptimal lytic efficiency, and the absence of lysis was recorded as zero lytic activity, following established EOP based phage efficacy assessment criteria (Panteleev et al., 2025).

Based on these EOP results, binary HR matrices were constructed by assigning a value of 1 to phage-strain combinations exhibiting productive lytic activity and 0 to those showing no detectable lysis. These HR matrices were used as input for the computational optimization of phage cocktail composition.

Phage cocktail optimization was performed using the optimal set selection strategy described by Díaz-Galián et al. (2022), implemented through the ExhaustiveSearch algorithm of the PhageCocktail package within the R statistical environment (R Core Team, 2025). To avoid unnecessarily complex formulations and based on previous systematic analyses indicating that phage cocktails composed of a limited number of well characterized phages achieve an effective balance between host specificity, antibacterial potency, and formulation stability, the maximum size of the phage cocktail was constrained to seven phages (Molina et al., 2022).

Phages selected by the computational optimization algorithm for cocktail formulation were subsequently subjected to more extensive phenotypic and genotypic characterization, together with a detailed evaluation of their efficacy against clinical *E. faecalis* strains.

### 2.6 Genomic characterization

Phage DNA was extracted from previously purified lysates. Aliquots of 500 μL of each lysate were treated with nucleases to remove residual host DNA and RNA. To this end, each sample was treated with 1.25 μL of DNase I (Applied Biosystems), along with 1.25 μL of RNase A (Omega Biotek), and incubated at 37 °C for 1 h 30 min. The nuclease reaction was stopped by adding 25 μL of 0.4 M EDTA, followed by incubation at 75 °C for 1 h. Subsequently, 20 μL of proteinase K (20 mg/mL) was added, and samples were incubated at 55 °C for 30 min. DNA was then purified using the Quick-DNA/RNA Viral Kit (Zymo Research) according to the manufacturer’s instructions.

Phage genomic sequencing was performed using Oxford Nanopore Technologies. Basecalled FASTQ reads were processed independently for each phage isolate. Read quality was assessed with NanoPlot v1.46.2 to inspect read length distribution, read quality scores and overall sequencing performance (De Coster and Rademakers, 2023). Reads were quality-filtered with Chopper v0.11.0 using a minimum read length of 500 bp and a minimum mean Phred quality score of 9 (De Coster and Rademakers, 2023). Filtered reads were assembled *de novo* with Flye v2.9.5 using the nano-hq option and five internal polishing iterations (Kolmogorov et al., 2019). Draft assemblies were further polished through five rounds of Racon v1.5.0 after read mapping with minimap2 v2.30 using the map-ont preset (Vaser et al., 2017; Li, 2018), followed by final polishing with Medaka v2.2.1 using the r941_min_sup_g507 model (Lee et al., 2021). Medaka-polished sequences were retained as the final assemblies for downstream analyses.

Genome quality and completeness were assessed using CheckV v1.0.3 with the CheckV database v1.5 and the end-to-end workflow (Nayfach et al., 2021). Structural and functional annotation was performed with Pharokka v1.8.1, with coding sequences predicted using PHANOTATE as implemented within Pharokka (McNair et al., 2019; Bouras et al., 2023). Annotated genomes were inspected to identify functional modules associated with DNA packaging, morphogenesis, host-cell lysis, DNA metabolism and other phage-related functions.

Taxonomic clustering of the phage genomes was performed through gene-sharing network analysis using vConTACT2 v0.11.3 (Jang et al., 2019). A custom reference database was generated from INPHARED by filtering entries to retain only phages reported to infect *Enterococcus* hosts (Cook and Hynes, 2025). The resulting network and genome overview table were inspected in Cytoscape v3.10.4 (Shannon et al., 2003), and the final figure was generated in R using the graph package.

For the six phages selected for the optimized cocktail (CKT6), taxonomic assignment at the genus and species levels was further assessed using taxMyPhage through the PhageCompass web service (Millard et al., 2025). Comparative genomic visualization was performed using clinker against RefSeq reference genomes representative of the corresponding predicted genera to assess genome organization and conserved functional modules (Gilchrist and Chooi, 2021). Intergenomic similarity was further assessed using VIRIDIC to calculate pairwise nucleotide-based genomic similarity, and using VirClust to calculate pairwise proteome-based similarity through shared protein clusters (PCs), both against RefSeq reference genomes representative of the corresponding predicted genera.

Genomic safety and lifestyle prediction were assessed using complementary in silico tools. PhageLeads was used to evaluate therapeutic suitability and screen for markers associated with temperate lifestyle, antimicrobial resistance and virulence (Yukgehnaish et al., 2022). Additional screening for antimicrobial resistance, virulence-associated and plasmid-related sequences was performed using ABRicate (Seemann, 2016). Phage lifestyle was further evaluated using PhaTYP as implemented in the PhaBOX platform (Shang et al., 2023a, b). Tail-associated genomic regions were inspected to identify coding sequences (CDS) potentially involved in host recognition, and putative depolymerase enzymes were predicted using the DePolymerase Predictor (DePP, web version 1.0.0), considering proteins with a predicted probability greater than 90% as potential depolymerases (Magill and Skvortsov, 2023).

### 2.7 Phenotypic characterization of selected cocktail phages

Plaque morphology was recorded for each phage on its corresponding propagation strain, and plaque diameter was quantified (n = 10 plaques per phage). Virions were examined by transmission electron microscopy (TEM) at the Centro de Investigación Príncipe Felipe (CIPF, Valencia, Spain) using a Tecnai G2 Spirit BioTWIN microscope operated at 120 kV. Lysates (10^8^–10^10^ PFU/mL) were first fixed in 2% (wt/vol) paraformaldehyde in aqueous solution, and 10 μL aliquots were applied onto carbon coated copper grids. After a 15 min adsorption period, grids were subjected to glow discharge at 7.2 kV, rinsed for 2 min in 0.1 M PBS, and post fixed in 1% (wt/vol) glutaraldehyde. Samples were negatively stained with 1% (wt/vol) uranyl acetate and subsequently contrasted in 1% (wt/vol) methyl cellulose for 30 s before air drying and storage until imaging. Phage morphotypes were assigned on the basis of virion architecture in TEM micrographs and classified according to current International Committee on Taxonomy of Viruses (ICTV) recommendations.

### 2.8 pH and temperature stability

Phage stability was evaluated under different pH and temperature conditions. For pH stability assays, high titer phage suspensions (>10^8^ PFU/mL) were diluted 1:10 in buffered solutions adjusted to pH 2–12 and incubated at 37.5 °C for 2 h. Phage viability was determined by serial dilution and quantified using the drop plate method. Phages incubated at pH 7 were used as controls to calculate survival percentages. All assays were performed in triplicate (Torres-Boncompte et al., 2025).

Thermal stability was assessed by incubating 1:10 dilutions of phage stocks (>10^8^ PFU/mL) in LB broth at 30–80 °C for 30 and 60 min. Following heat exposure, phage titers were determined by serial dilution and drop plate enumeration. Samples stored at 4 °C and −80 °C were used as controls to establish baseline stability (Torres-Boncompte et al., 2025).

### 2.9 Kinetics of infection of individual selected phages

Infection kinetics were assessed as described by Alrafaie et al. (2024) with slight modifications. In 96-well plates, 160 μL of LB together with 20 μL of the exponential-phase of host bacterial suspension (OD_600_ = 0.4) and 20 μL of phage suspension were added to achieve multiplicity of infection (MOI) of 10, 1, 0.1 and 0.01. OD_600_ measurements were taken every 5 min up to 12 h at 37 °C with continuous shaking using a microplate spectrophotometer (Multiskan SkyHigh, Thermo Fisher Scientific™). All the values obtained were later corrected with blank values.

### 2.10 One-step growth and burst size analysis

To estimate the latent period and burst size of the six phages selected for the CKT6 cocktail, one-step growth curves were performed based on the protocol previously reported by Torres-Boncompte et al. (2025). Briefly, host bacterial cultures were grown to exponential phase (OD₆₀₀ = 0.4) and inoculated with each phage at a multiplicity of infection (MOI) of 0.1, allowing adsorption for 10 min at 37 °C. Following centrifugation to remove non-adsorbed phage particles, infected cells were resuspended in fresh LB broth. Aliquots were collected at defined time points and phage titers were determined by the double-layer agar (DLA) technique. Latent period was defined as the interval elapsed between infection and the beginning of the first progeny burst, whereas burst size was estimated by dividing the phage titer at the plateau phase by the number of bacterial cells initially infected. Aliquots were collected every 10 min over a 100 min period, and phage titers were determined by the double-layer agar (DLA) technique. Latent period was defined as the interval elapsed between infection and the beginning of the first progeny burst. Burst size was calculated according to the following formula: Burst size = (average phage titer at the plateau phase − average phage titer before the burst) / (number of infected bacterial cells); where the number of infected bacterial cells was estimated as the phage inoculum administered (MOI × initial bacterial concentration) minus the titer of non-adsorbed phages recovered in the supernatant after the 10-min centrifugation step. All titer values used in this calculation were expressed in PFU prior to log₁₀ (PFU/mL) transformation for graphical representation. One-step growth curve experiments were performed in biological triplicate (*n* = 3) for each phage, and results are expressed as mean ± standard deviation.

**Figure 7:**
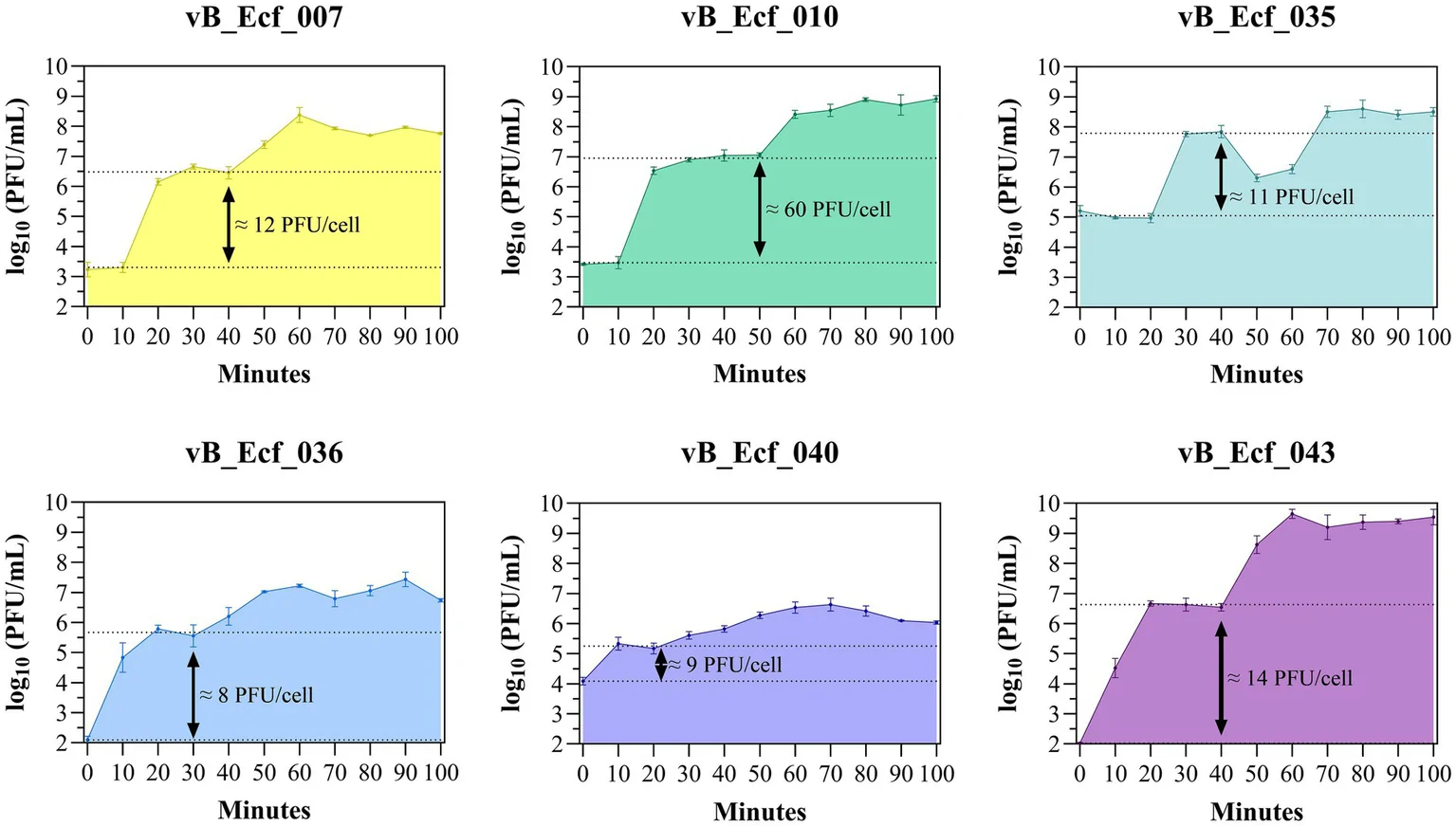
One-step growth curves of the six phages selected for the CKT6 cocktail. Phage titers (log₁₀ PFU/mL) are shown 100 min post-infection. Dotted lines indicate the initial and final titer plateaus used to estimate burst size (PFU/cell), shown for each phage.

### 2.11 *In vitro* efficacy of the optimized cocktail

The *in vitro* lytic activity of the optimized phage cocktail was assessed for all clinical *E. faecalis* strains using the previously described kinetic infection assay at a MOI of 1, including untreated bacterial controls. Lytic activity was determined by comparing the growth kinetics of phage-treated cultures with controls. All assays were performed at least in triplicate to ensure reproducibility.

### 2.12 Statistical analysis

Phage-host interaction analyses were performed using the EOP matrix obtained from host range assays. Phage-strain combinations with EOP values ≥ 0.5 were considered positive productive interactions. The association between genotypic relatedness and EOP-based infectivity profiles was assessed using a Mantel test with the vegan package. The genotypic distance matrix was compared with the phenotypic Bray–Curtis dissimilarity matrix calculated from quantitative EOP values. Spearman’s rank correlation coefficient was used, and significance was assessed using 9,999 unrestricted permutations (R Studio v.2026.04.).

Differences in lysis patterns among cocktails were analysed using cumulative link mixed models, considering lysis outcome as an ordinal variable (P < T < C). Complete lysis was further analysed as a binary outcome (C *v*s. T/P) using mixed-effects logistic regression, with cocktail formulation as a fixed effect and bacterial strain as a random effect. Pairwise comparisons were performed using estimated marginal means with Holm correction.

## 3 Results

### 3.1 Phage diversity and distribution across viral clusters

Extensive phage hunting was performed on poultry farm samples collected over a one-year period, using 51 *E. faecalis* strains as primary hosts. A total of 38 *E. faecalis* bacteriophages with demonstrated lytic activity were isolated from 880 pooled samples (equivalent to 8,800 individual field samples). Lytic activity characterization using the DLA method confirmed that the complete phage collection was capable of producing lytic activity against 94.12% (*n* = 48/51) of the tested *E. faecalis* strains, with only three strains showing either turbid lysis or no productive lysis (Supplementary Figure 1).

The 38 isolated phages were successfully sequenced and characterized. Combined gene-sharing network analysis and genome-based taxonomic assignment supported the classification of the phage collection into three predicted viral genera: *Saphexavirus* (*n* = 31; 81.58%), *Efquatrovirus* (*n* = 4; 10.53%) and *Kochikohdavirus* (*n* = 3; 7.89%) (Figure 1).

**Figure 1:**
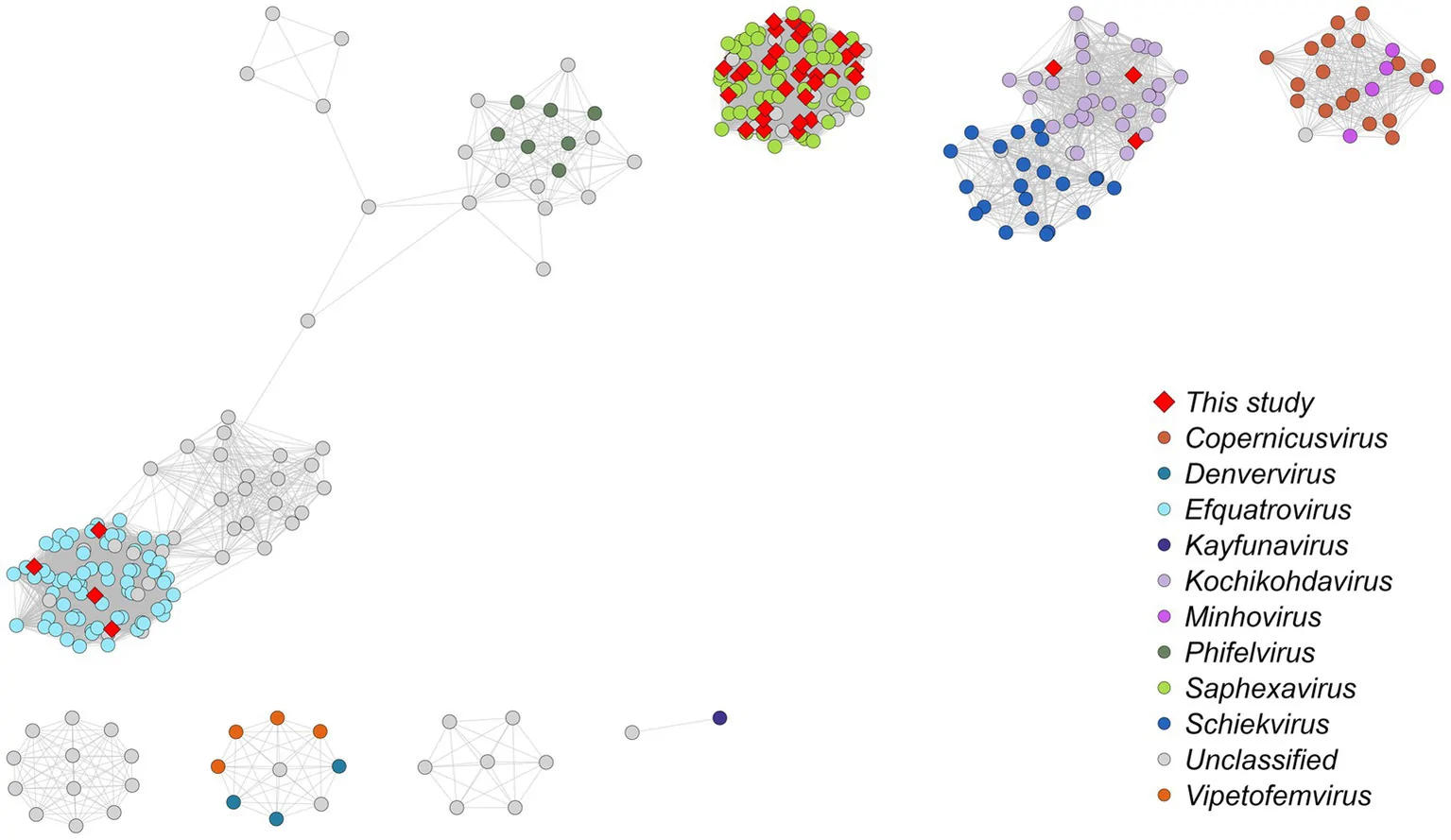
Gene-sharing network analysis of *Enterococcus* phages isolated in this study and related reference phages. The network was generated using vConTACT2 based on shared gene content. Red diamonds indicate phages isolated in this study, circles represent reference phages, node colours indicate genus-level taxonomic assignments, and grey nodes correspond to unclassified phages.

### 3.2 Phage host range and EOP-based infectivity clustering

EOP analysis was used to determine the productive infectivity of the 38-phage collection against the 51 *E. faecalis* strains. Although phage-host interactions were highly specific, the collection showed broad overall coverage, producing complete lysis in 48 of the 51 tested strains (94.12%) under the conditions evaluated (Figure 2A). The remaining three strains (T103, T133, and T196) exhibited only partial clearing zones, as no individual phage within the collection was capable of producing complete lysis on these hosts. Consequently, these strains were excluded from the EOP analysis. The integrated EOP heatmap and genomic relatedness dendrogram revealed heterogeneous infectivity profiles across the bacterial panel (Figure 2A). Some genomically related phages showed partially overlapping EOP-based profiles, whereas others differed in their infectivity patterns despite close genomic relatedness. Conversely, phages located in distinct genomic branches displayed complementary host-range profiles. This visual pattern was supported by Mantel analysis, which showed a significant positive association between the genotypic distance matrix and the EOP-based Bray–Curtis dissimilarity matrix (r = 0.5215; *p* = 0.0001). Thus, genomic relatedness was moderately associated with infectivity profiles, but genome-wide similarity alone did not fully explain phage host range, suggesting that genome-wide similarity alone is insufficient to predict host range, reinforcing the relevance of phenotypic screening for accurate cocktail design.

**Figure 2:**
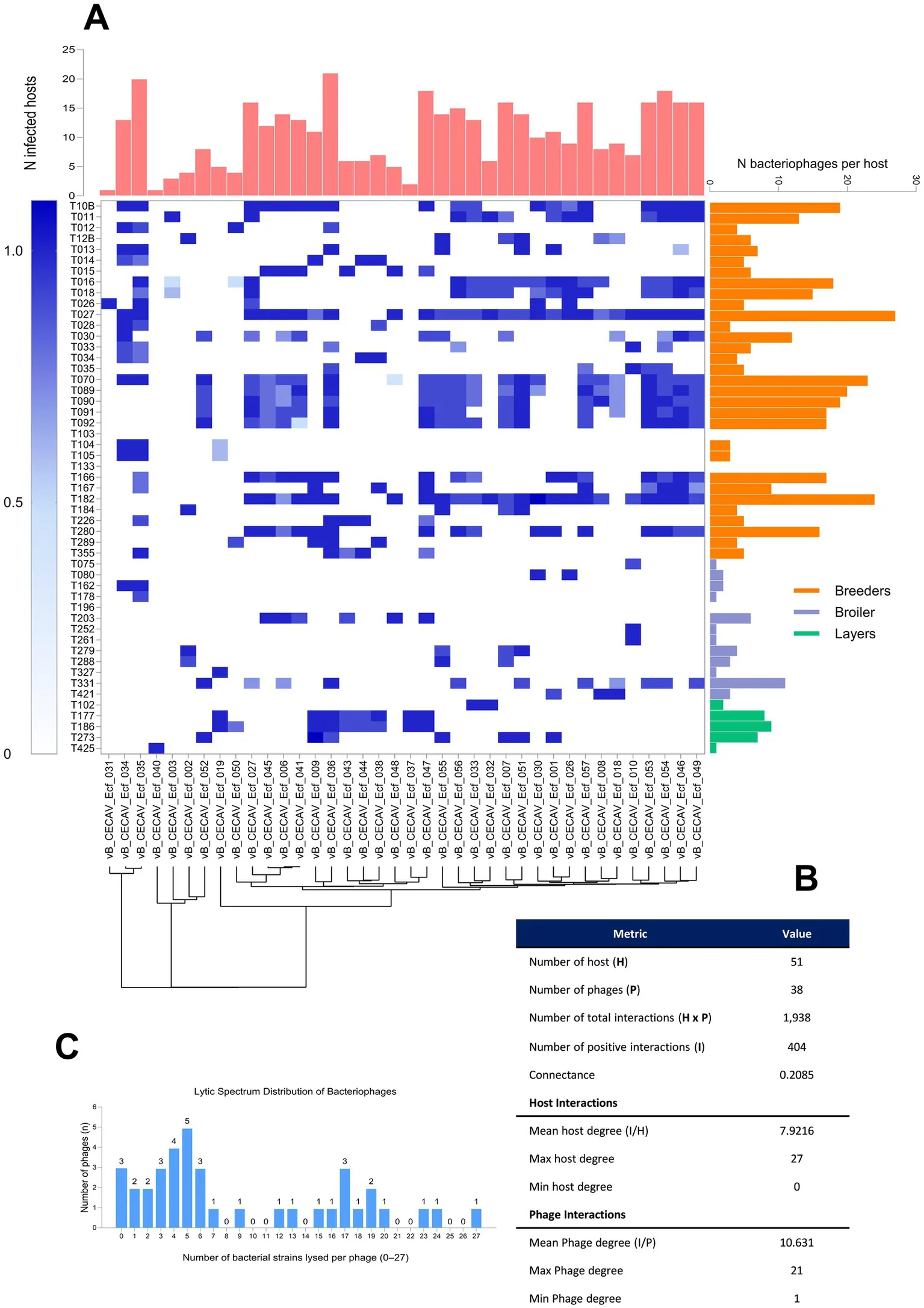
Host range, interaction metrics and lytic spectrum of the isolated *E. faecalis* phage collection. **(A)** EOP-based host range heatmap showing the infectivity profiles of the 38 bacteriophages against the 51 *E. faecalis* strains. Blue intensity represents normalized EOP values, while white indicates no detectable productive infection. The dendrogram shows clustering of phages according to their EOP-based infectivity profiles. Bar plots indicate the lytic spectrum of each phage and the susceptibility of each bacterial strain, with strains grouped according to poultry production type. **(B)** Summary metrics of the phage-host interaction matrix. **(C)** Distribution of the lytic spectrum of the phage collections.

When strains were grouped according to their production origin, phage coverage was consistently high across the different poultry categories. All layer-associated strains were completely lysed by at least one phage (5/5; 100%), while high coverage was also observed among breeder-associated strains (31/33; 93.94%) and broiler-associated strains (12/13; 92.31%).

The interaction metrics further highlighted the structured and specific nature of the phage-host network (Figure 2B). From a total of 1,938 possible phage-host combinations, 404 were classified as positive productive interactions, resulting in a connectance of 0.2085. This relatively low connectance indicates that, although the collection achieved broad cumulative coverage, individual phage-host interactions remained highly specific. On average, each bacterial strain was infected by 7.92 phages, although host susceptibility varied markedly, ranging from strains with no productive interactions to strains infected by up to 27 phages. Similarly, each phage infected an average of 10.63 bacterial strains, with a wide variation in lytic spectrum among phages.

This variability was also reflected in the distribution of phage lytic spectra (Figure 2C). Most phages showed narrow to intermediate host ranges, whereas a smaller subset displayed broader infectivity profiles. Eleven phages (11/38; 28.95%) showed a broad host range, defined here as productive infection of 15 or more *E. faecalis* strains. Among them, vB_Ecf_035 and vB_Ecf_036 phages exhibited the widest spectra, infecting 21 and 20 strains, respectively, followed by vB_Ecf_047 and vB_Ecf_054, which infected 18 strains each. Collectively, these broad-host-range phages covered 40 of the 51 tested strains (78.43%), supporting their relevance as key candidates for rational multi-phage cocktail design.

### 3.3 Rational selection of optimized cocktail candidates

The PhageCocktail computational tool enabled the identification of an optimized broad-spectrum combination composed of five (CKT5: vB_Ecf_007, vB_Ecf_010, vB_Ecf_035, vB_Ecf_036, and vB_Ecf_043), six (CKT6: vB_Ecf_007, vB_Ecf_010, vB_Ecf_035, vB_Ecf_036, vB_Ecf_040, and vB_Ecf_043) and seven (CKT7: vB_Ecf_001, vB_Ecf_010, vB_Ecf_035, vB_Ecf_036, vB_Ecf_040, vB_Ecf_043, and vB_Ecf_055) phages, designed to achieve extensive coverage against the total analysed strain collection. CKT7 and CKT5 were able to lyse 88% of the strains (45/51) whereas CKT6 was able to lyse 92% of the strains (47/51). Mixed-effects logistic regression showed no statistically significant differences among cocktails after Holm correction (all adjusted *p* > 0.05). However, CKT6 showed the most favourable descriptive performance, with approximately 2.26-fold higher odds of complete lysis than CKT5 and 1.33-fold higher odds than CKT7. Given this trend, together with its broader formulation than CKT5 and lower complexity than CKT7, CKT6 was selected as the optimized formulation for downstream characterization and *in vitro* validation.

### 3.4 Phage genomic characterization and annotation

Full genomic characterization was performed for the six phages selected for the optimized CKT6 cocktail (vB_Ecf_007, vB_Ecf_010, vB_Ecf_035, vB_Ecf_036, vB_Ecf_040, and vB_Ecf_043). All six phages were predicted to have a virulent lifestyle. In addition, no antimicrobial resistance genes, virulence-associated genes, plasmid-related sequences or other undesirable genetic determinants were detected (Table 1).

**Table 1:** Genomic and taxonomic characteristics of CKT6 phages.

Feature	vB_Ecf_007	vB_Ecf_010	vB_Ecf_035	vB_Ecf_036	vB_Ecf_040	vB_Ecf_043
Genome size (Kb)	58	57	144	59	41	57
GC content (%)	39.6	39.8	35.9	39.9	34.9	39.8
Coding sequences (CDS)	115	94	207	98	72	94
tRNAs/pseudogenes	1	1	5	1	0	1
Hypothetical proteins (%)	63	61	56	55	57	62
Accession number	PZ295607.1	PZ295608.1	PZ295609.1	PZ295610.1	PZ400691.1	PZ295606.1
Class	Caudoviricetes	Caudoviricetes	Caudoviricetes	Caudoviricetes	Caudoviricetes	Caudoviricetes
Family	Not defined	Not defined	Herelleviridae	Not defined	Not defined	Not defined
Genus	Saphexavirus	Saphexavirus	Kochikohdavirus	Saphexavirus	Efquatrovirus	Saphexavirus
Predicted lifestyle	Virulent	Virulent	Virulent	Virulent	Virulent	Virulent
AMR/virulence/plasmid markers	Not detected	Not detected	Not detected	Not detected	Not detected	Not detected

Genome sizes ranged from 41,256 bp for vB_Ecf_040 to 143,904 bp for vB_Ecf_035, with GC contents between 34.9 and 39.9%. The number of predicted coding sequences varied among phages, from 72 CDS in vB_Ecf_040 to 207 CDS in vB_Ecf_035. Hypothetical proteins represented a high proportion of the predicted proteomes, ranging from 55 to 63% (Table 1).

Genome-based taxonomic assignment classified all six phages within the class *Caudoviricetes*. At the genus level, four phages were assigned to *Saphexavirus* (vB_Ecf_007, vB_Ecf_010, vB_Ecf_036, and vB_Ecf_043) whereas vB_Ecf_035 was assigned to *Kochikohdavirus* and vB_Ecf_040 to *Efquatrovirus* (Table 1).

Comparative genomic visualization using clinker was performed using RefSeq reference genomes from the corresponding predicted genera. The six CKT6 phages displayed modular genome organization, including regions associated with DNA packaging, head and tail morphogenesis, host-cell lysis, and DNA metabolism, with variation observed mainly in structural and tail-associated regions (Figure 3). Intergenomic similarity analyses using VIRIDIC (Figure 4) and VirClust (Supplementary Figure 2) further supported the genus-level taxonomic assignments obtained through gene-sharing network analysis and taxMyPhage, with nucleotide and proteome-based similarity values consistent with the predicted genus boundaries for each phage.

**Figure 3:**
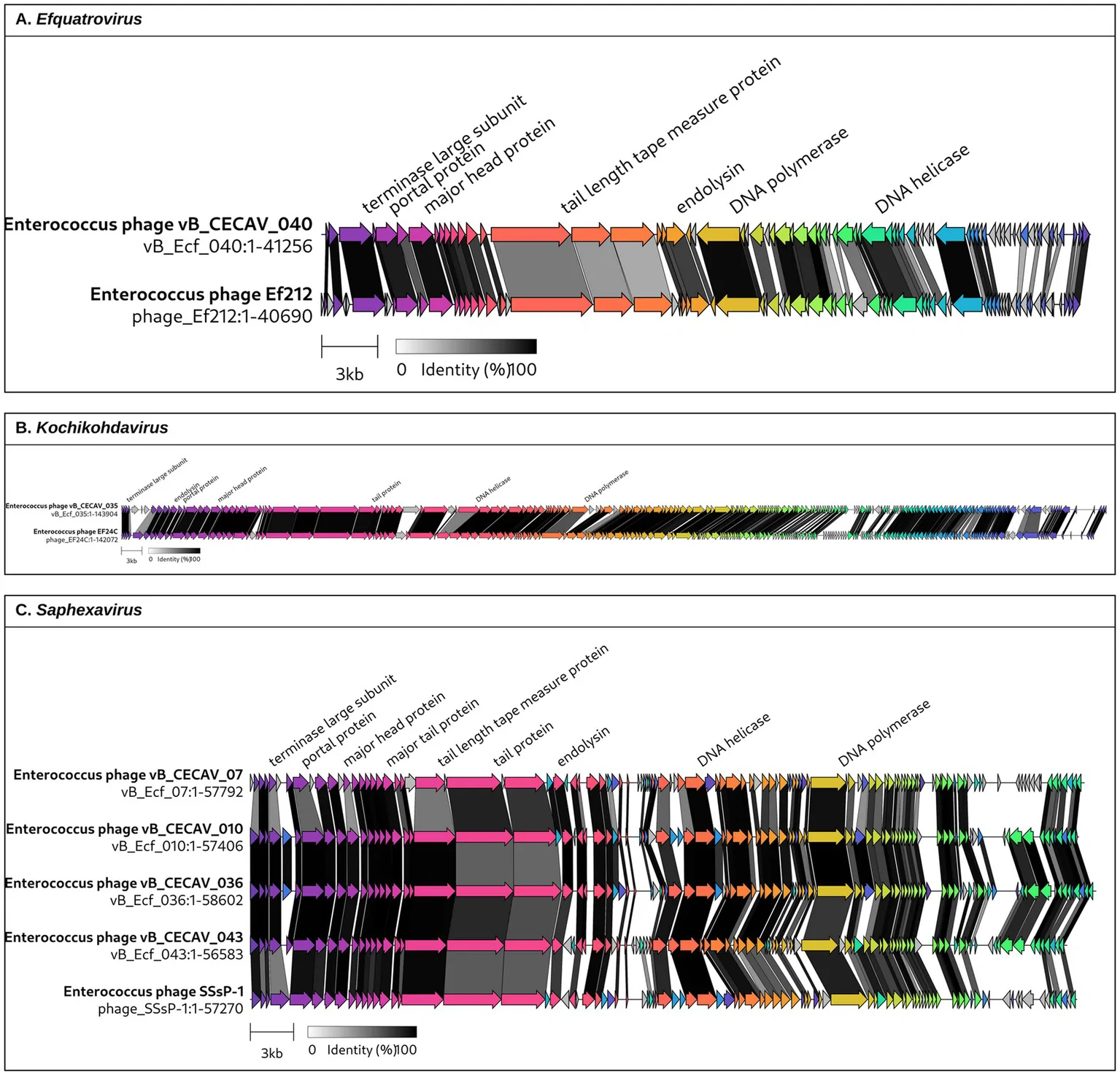
Comparative genomic organization of CKT6 phages and RefSeq genomes from the corresponding predicted genera. Arrows represent predicted coding sequences coloured according to functional annotation, and shaded regions indicate homologous genomic regions between phages.

**Figure 4:**
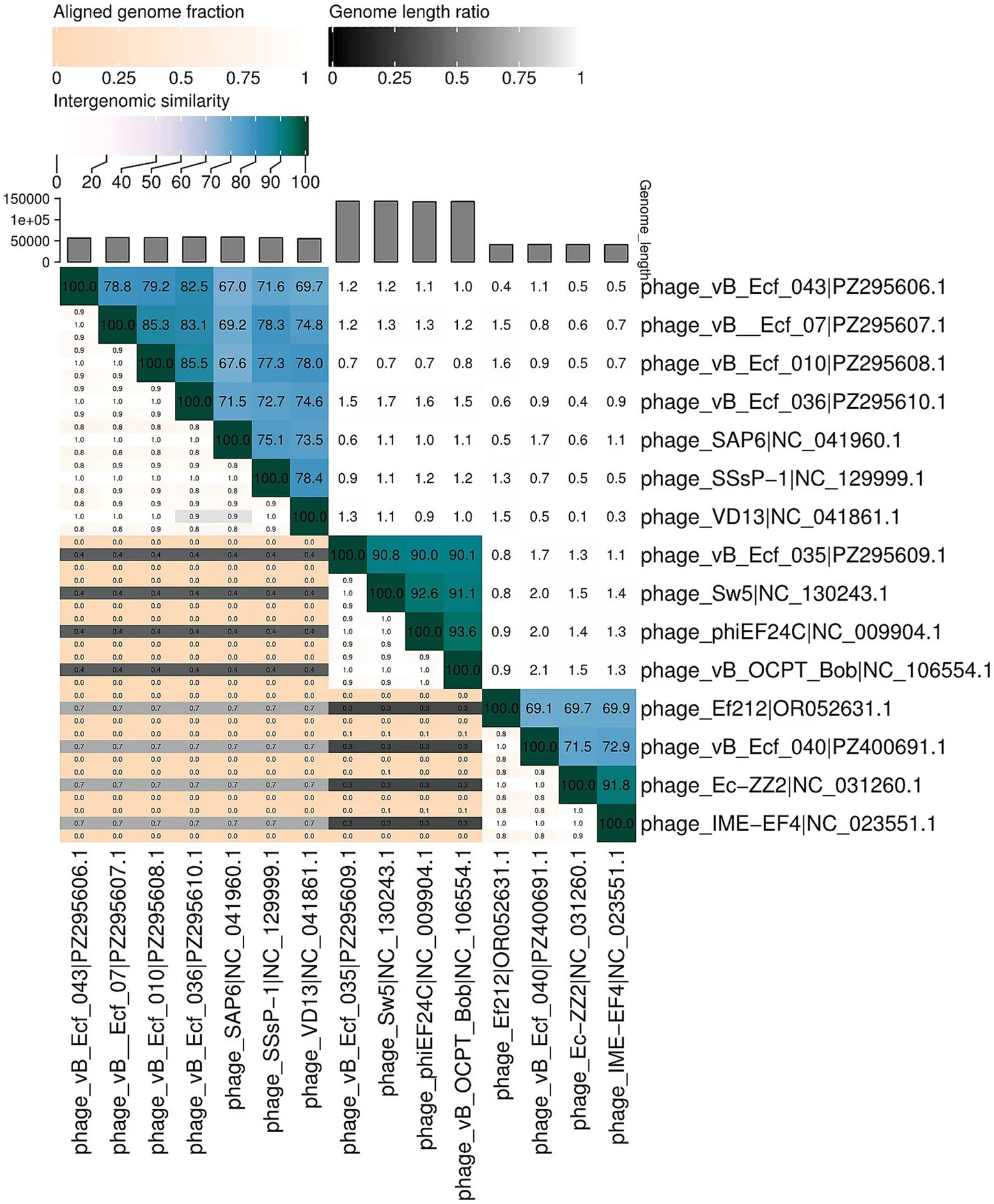
Intergenomic similarity heatmap generated by VIRIDIC. The matrix shows pairwise genomic similarity between the studied bacteriophages and RefSeq genomes from the corresponding predicted genera. Values above 70% indicate genus-level relatedness.

### 3.5 Putative depolymerase CDS in CKT6 phages

DePP analysis identified CDS with a predicted probability greater than 90% of encoding putative depolymerases in tail-associated genomic regions of the six CKT6 phages (Table 2). These CDS were annotated as tail proteins, Tal-like tail tip proteins, tail fiber proteins, Dit-like distal tail proteins, minor tail proteins, virion structural proteins, or hypothetical proteins. vB_Ecf_035 contained the highest number of DePP-predicted candidate depolymerase CDS, including a tail protein with lysin activity and a tail fiber protein.

**Table 2:** Predicted depolymerases in CKT6’s phages by DePP.

Phage	CDS	Locus	Start	End	Strand	Function	Product
vB_Ecf_007	25	cecavEcc07_025	13.638	17.363	+	Tail	Tail protein
	27	cecavEcc07_027	17.645	20.380	+	Head and packaging	Tail tip protein Tal
vB_Ecf_010	20	cecavEcc010_020	14.253	18.245	+	Tail	Tail protein
	21	cecavEcc010_021	18.258	21.284	+	Head and packaging	Tail tip protein Tal
vB_Ecf_035	32	cecavEcc035_032	25.910	29.095	+	Tail	Tail protein with lysin activity
	33	cecavEcc035_033	29.181	34.658	+	Tail	Tail fiber protein
	34	cecavEcc035_034	34.758	37.169	+	Tail	Tail protein
	44	cecavEcc035_044	44.289	47.753	+	Unknown function	Hypothetical protein
vB_Ecf_036	21	cecavEcc036_021	14.258	18.253	+	Tail	Tail protein
	22	cecavEcc036_022	18.265	21.570	+	Head and packaging	Tail tip protein Tal
vB_Ecf_040	16	cecavEcc040_016	13.409	15.490	+	Tail	Distal tail protein Dit
	17	cecavEcc040_017	15.503	17.815	+	Tail	Minor tail protein
vB_Ecf_043	19	cecavEcc043_019	13.631	17.623	+	Tail	Tail protein
	20	cecavEcc043_020	17.635	20.862	+	Head and packaging	Virion structural protein

### 3.6 Morphological and phenotypic characterization of cocktail phages

TEM analysis revealed distinct morphological features among the six bacteriophages, which belong to three different genera (Figure 5). Four phages (vB_Ecf_007, vB_Ecf_010, vB_Ecf_036, and vB_Ecf_043) displayed the characteristic prolate head morphology of the genus *Saphexavirus*, with a long, flexible, non-contractile tail. These phages exhibited elongated capsids with a mean head length ranging from 110.2 ± 1.4 nm (vB_Ecf_036) to 117.3 ± 2.0 nm (vB_Ecf_010) and a mean head width ranging from 41.0 ± 0.3 nm (vB_Ecf_036) to 46.2 ± 1.1 nm (vB_Ecf_007). Tail lengths for *Saphexavirus* phages ranged from 136.9 ± 1.4 nm (vB_Ecf_043) to 147.6 ± 2.2 nm (vB_Ecf_036), with tail widths between 10.8 ± 0.7 nm (vB_Ecf_010) and 12.3 ± 0.4 nm (vB_Ecf_007).

**Figure 5:**
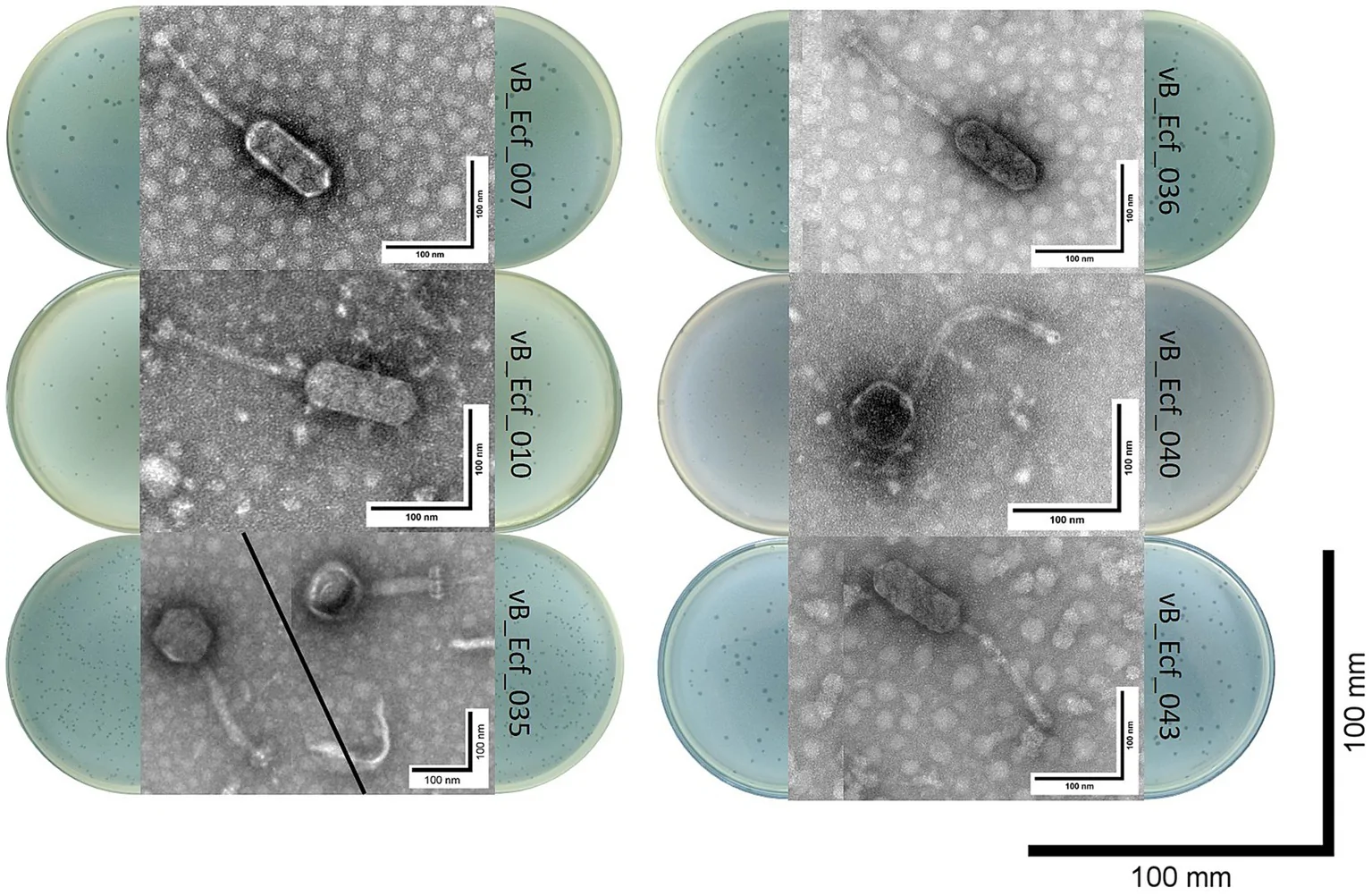
Plaque morphology and representative TEM micrographs of the six bacteriophages comprising CKT6. TEM scale bars indicate 100 nm. The scale bar in the bottom-right corner of the Petri dish images indicates 100 mm.

Phage vB_Ecf_035 was classified as a member of the genus *Kochikohdavirus*, exhibiting an isometric icosahedral head (94.0 ± 3.0 nm in diameter) and a contractile tail characteristic of this genus. The extended tail measured 180.2 ± 2.3 nm in length and 19.9 ± 1.7 nm in width. Upon contraction, the tail sheath shortened to 127.5 ± 1.7 nm (29.2% reduction in length, contraction ratio: 1.41×) while increasing in width to 26.8 ± 0.8 nm, reflecting the conformational rearrangement of the tail sheath that drives genome injection into the host cell.

Phage vB_Ecf_040 displayed the morphology characteristic of the genus *Efquatrovirus*, with an isometric icosahedral capsid (66.3 ± 2.1 nm in diameter) and a long, flexible, non-contractile tail (215.8 ± 2.0 nm in length, 10.3 ± 0.8 nm in width). This phage presented the smallest capsid among all six phages and the longest tail.

Regarding pH stability, it ranged between pH 3 and 11, with maximal stability from pH 5 to 9 (Figure 6A). All isolates were completely inactivated at pH 2, indicating high sensitivity to extreme acidic conditions. At pH 3, partial infectivity was retained, with vB_Ecf_010 showing the highest acid tolerance. Viability markedly improved at pH 4, suggesting this value as a lower stability threshold. All phages displayed optimal stability from pH 5 to 9, with complete preservation of infectivity at pH 7 and consistently high viability under neutral to mildly alkaline conditions. A gradual decrease was observed at pH 10 and pH 11, although most isolates still retained substantial infectivity. Complete inactivation occurred at pH 12, confirming sensitivity to extreme alkaline conditions.

**Figure 6:**
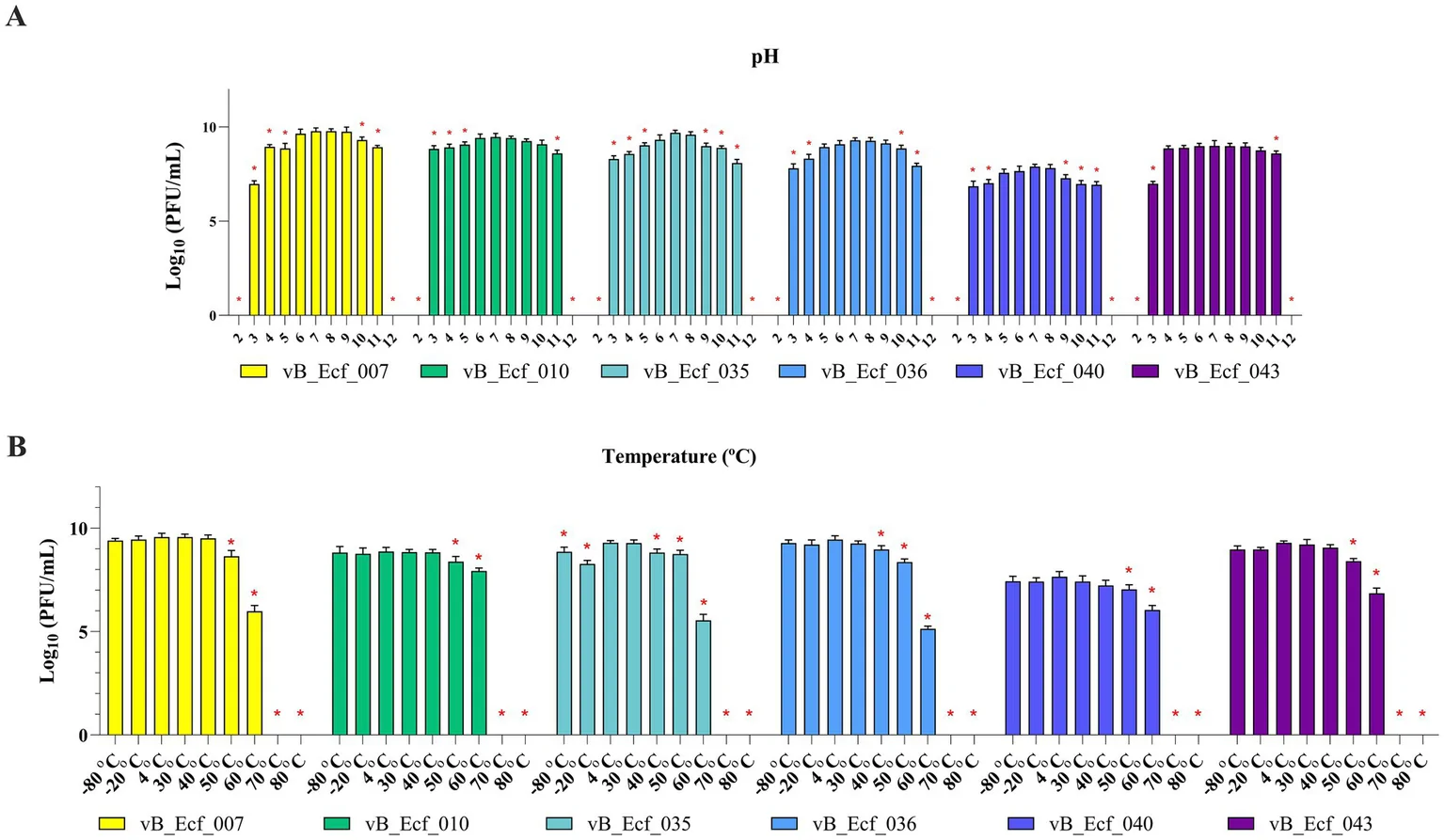
pH **(A)** and thermal **(B)** stability of *E. faecalis* bacteriophage isolates. Bar charts showing mean phage viability after 60 min of exposure to each temperature or after 2 h of exposure to each pH condition. with error bars representing standard deviation across biological replicates. Asterisks (*) denote statistically significant differences compared with the control condition (pH 7 for pH stability or 4 °C for thermal stability).

No significant differences in phage viability were detected between 30 and 60 min of exposure across all temperatures evaluated, therefore, only the results obtained after 60 min of exposure period are presented below (Figure 6B). The thermal stability assays showed that the six-bacteriophages remained stable under refrigeration and frozen storage conditions, with 100% viability at 4 °C and high infectivity at −80 °C and −20 °C. Similarly, viability remained high at 30 °C and 40 °C, indicating good tolerance to ambient and moderately elevated temperatures. A progressive reduction in infectivity was observed from 50 °C onwards. At 50 °C, all isolates retained relatively high viability, with vB_Ecf_010 and vB_Ecf_035 showing the greatest stability. However, at 60 °C, viability declined markedly and varied among isolates, with vB_Ecf_010 displaying the highest residual infectivity and vB_Ecf_036 the lowest. Complete inactivation was observed at 70 °C and 80 °C for all phages. Stability values with the corresponding standard deviations across biological replicates are shown in Figure 6 for both pH and temperature conditions; asterisks (*) indicate statistically significant differences relative to the control condition (pH 7 or 4 °C, respectively).

### 3.7 One-step growth kinetics and burst size of CKT6 phages

One-step growth curves were determined for the six phages included in the CKT6 cocktail to characterize their latent period and burst size (Figure 7). All six phages displayed a latent period of approximately 20 min, accounting for the 10 min required to enter the exponential growth phase and an additional 10 min allotted to ensure proper attachment, and were followed by a clear rise phase reflecting the release of progeny virions. Burst size varied considerably among phages, ranging from approximately 8 PFU/cell for vB_Ecf_036 to approximately 60 PFU/cell for vB_Ecf_010. Phages vB_Ecf_007, vB_Ecf_035, vB_Ecf_040, and vB_Ecf_043 showed intermediate burst sizes of approximately 12, 11, 9, and 14 PFU/cell, respectively. vB_Ecf_010 was clearly the most productive phage of the cocktail, with a burst size roughly five- to seven-fold higher than the other five phages, while vB_Ecf_036 showed the lowest replicative yield.

### 3.8 The optimized CKT6 cocktail shows broad *in vitro* activity against clinical poultry isolates

The lytic kinetics of the six phages included in the selected cocktail are shown in Supplementary Figure 3. All phages suppressed or delayed bacterial growth compared with the untreated control, with a clear MOI-dependent effect. Higher MOIs (10 and 1) generally produced faster and stronger reductions in OD, whereas lower MOIs (0.1 and 0.01) were frequently associated with partial bacterial regrowth.

Cocktail CKT6 showed broad lytic activity against the *E. faecalis* strain panel at MOI 1, with 47/51 strains (92.16%) displaying a biphasic kinetic profile characterized by an initial growth phase followed by a marked decrease in normalized OD (Figure 8A). Four strains, T102, T103, T327, and T421, were not effectively controlled by the cocktail and maintained high OD values until the end of the assay. This lack of response was consistent with the absence of productive infectivity of the cocktail components against T103, T327, and T421 in previous EOP assays (see Figure 2), whereas T102 showed only moderate individual phage activity that did not translate into effective suppression in liquid culture. Conversely, T133 and T196 were lysed by the cocktail despite the absence of clearly effective individual activity in the initial screening. To assess whether OD_600_-based inhibition reflected a reduction in viable bacteria, CFU enumeration was performed in a subset of strains selected as a representative approximation of the different kinetic profiles observed across the collection. These six strains were chosen to illustrate the range of responses identified among the 51 tested isolates, including early (time < 2 h; *n* = 22), middle (time 2-3 h; *n* = 22) and late-phase (time >3 h; *n* = 3) responders, the discordant strains (*n* = 2), and the non-responder strains (*n* = 2) (Figure 8B).

**Figure 8:**
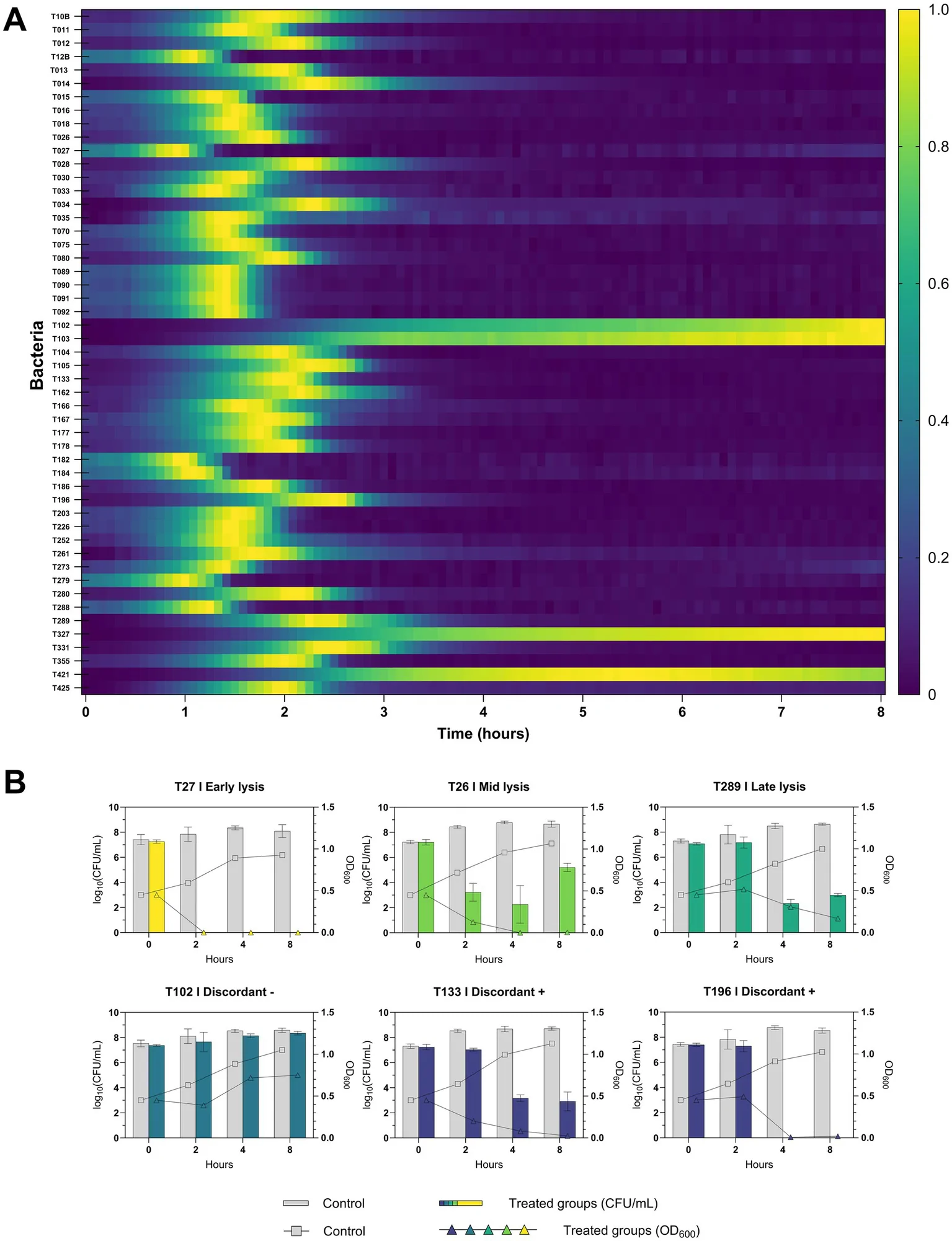
Lytic kinetics of cocktail CKT6 and association between phage interaction outcomes and early bacterial suppression. **(A)** Heat map showing normalised OD_600_ kinetics of 51 *Enterococcus* strains exposed to cocktail CKT6 at MOI 1 over 8 h. **(B)** Bacterial counts and OD_600_ profiles in representative strains exposed to CKT6. Bars indicate log_10_ CFU/mL, while superimposed lines indicate OD_600_ values.

In agreement with the OD₆₀₀ kinetics, CFU enumeration confirmed a marked reduction in viable bacterial counts across the subset of strains tested. CKT6 treatment resulted in no detectable viable recovery for the early-phase responder T27 at 8 h, consistent with a rapid and complete bactericidal effect; counts fell below the limit of detection of the assay (<100 CFU/mL). For T26, classified as a mid-phase responder, a substantial reduction in viable counts was observed, although residual recovery above the detection limit was detected at late time points, suggesting a slower but nonetheless significant lytic effect. The late responder T289 showed a marked reduction in recoverable counts, consistent with the delayed but effective lytic profile observed in the OD₆₀₀ kinetics. The discordant strains T133 and T196, which lacked clearly effective individual phage activity in the initial screening, also exhibited a strong reduction in viable counts upon CKT6 treatment, supporting the ability of the cocktail to control strains with limited individual phage susceptibility, likely through synergistic or complementary activity among cocktail components. In contrast, T102 showed only partial reduction in viable counts, consistent with its poor suppression profile in liquid culture and the moderate individual phage activity previously recorded.

## 4 Discussion

*Enterococcus faecalis* is an opportunistic pathogen of One Health relevance. It causes difficult-to-treat human infections and displays both intrinsic and acquired antimicrobial resistance, while poultry-associated enterococci may further contribute to the maintenance and dissemination of resistance determinants along the food chain (Pedersen et al., 2008; Poulsen et al., 2012; Bortolaia et al., 2016; Rodríguez-Lucas and Ladero, 2023; Said et al., 2024). In this context, phages targeting MDR *E. faecalis* from poultry production could provide a complementary strategy to reduce bacterial reservoirs at the animal-production level.

The main contribution of this study is the development of a rational and reproducible approach for phage cocktail design against MDR poultry-associated *E. faecalis*. By coupling extensive field-derived phage isolation with systematic host-range characterization, quantitative infectivity metrics, genomic analysis and computational optimization, this work moves beyond empirical phage selection and provides a data-driven strategy to address the marked heterogeneity of *E. faecalis* populations in poultry production systems.

Here, we report, to our knowledge, one of the largest poultry-associated collections of *E. faecalis* phages described to date, including 38 fully sequenced phages evaluated against 51 clinical poultry isolates. Compared with previous poultry-related studies, which have generally described individual or small numbers of *E. faecalis* phages such as EFC1, EFP6, C-3 and G21-7, the predominance of *Saphexavirus* in our collection (31 out of 38) suggests a recurrent enrichment of this lineage among phages infecting avian-associated *E. faecalis* (Wang and Liu, 2022; Ahmad et al., 2024; Wang et al., 2024; Onaran Acar et al., 2026). However, the description of non-*Saphexavirus* poultry-associated phages indicates that this pattern reflects enrichment rather than exclusivity. These findings position poultry environments as valuable reservoirs of specialized phages for the rational design of targeted biocontrol strategies.

The high functional coverage achieved by the phage collection, with productive infection detected in 94.12% of the tested strains, highlights the value of field-derived phages as candidates for broad-spectrum biocontrol against poultry-associated *E. faecalis*. Importantly, this coverage was consistently high across poultry production systems, as all layer-associated strains were lysed by at least one phage, while high susceptibility was also observed among breeder and broiler-associated isolates. This is particularly relevant from an applied perspective, as an effective phage-based strategy for poultry production should not be restricted to a single production type, but should retain activity across the bacterial diversity encountered in different epidemiological and management contexts.

In parallel, the genomic diversity of the collection was partially reflected in its infectivity patterns, as Mantel analysis revealed a significant but moderate association between genomic relatedness and EOP-based host-range profiles. Thus, genetically related phages tended to behave similarly, but genome-wide similarity alone did not fully predict infectivity. This is consistent with the concept that phage host range is determined by multiple molecular interactions throughout the infection cycle, particularly receptor recognition, adsorption and post-adsorption compatibility (Hyman and Abedon, 2010; de Jonge et al., 2019). In tailed phages, receptor-binding proteins and tail-associated structures play a central role in host recognition and may strongly influence host specificity even among related phages (Le et al., 2013; Dunne et al., 2021). Therefore, our findings support a cocktail-design strategy that integrates genomic diversity with experimental infectivity data rather than relying solely on phage taxonomy or sequence similarity (Forti et al., 2018; Yang M. et al., 2020; Koncz et al., 2024).

A central aspect of this study was the rational design of the CKT6 cocktail. By integrating EOP-derived HR matrices with computational optimization, a broadly active formulation was obtained while avoiding unnecessary cocktail complexity. The subsequent characterization of the six selected phages further supports their suitability as biocontrol candidates. Their genomes lacked detectable antimicrobial resistance genes, virulence-associated genes or other undesirable genetic determinants, which is an essential safety criterion for phages intended for therapeutic or food-chain applications (Cook and Hynes, 2025). Comparative genomic analysis showed conserved modular organization within the predicted viral genera, while structural and tail-associated regions displayed variability among selected phages. This genomic variability is relevant for cocktail design because tail-associated proteins, including tail fibers and adsorption-related structures, are commonly involved in phage-host interactions and may contribute to differences in host range. The marked variability in burst size observed among the CKT6 phages, ranging from approximately 8 to 60 PFU/cell, further illustrates the heterogeneity in replicative fitness among cocktail components, which may influence their relative contribution to overall bactericidal activity in multi-phage formulations.

Putative depolymerase-associated activities were inferred from plaque morphology and tail-associated annotations. Phage-encoded depolymerases are known to degrade bacterial surface polysaccharides and extracellular polymeric substances, potentially improving phage access to bacteria embedded in structured biofilm matrices (Topka-Bielecka et al., 2021; Guo et al., 2023). The presence of candidate host-recognition and depolymerase-associated proteins is of interest in the context of *E. faecalis*, a species strongly associated with biofilm formation, persistence and increased antimicrobial tolerance (Dale et al., 2017; Lee, 2017; Moryl et al., 2023).

The performance of CKT6 in liquid culture further illustrates the complexity of phage-host interactions and highlights a critical methodological consideration for cocktail efficacy assessment. Although the cocktail showed broad *in vitro* activity, responses varied among strains, indicating that cocktail efficacy cannot be inferred solely from individual phage activity or from genomic similarity. Of note, OD-based kinetic monitoring alone may not fully capture the bactericidal outcome of phage treatment, a sharp decline in optical density is consistent with effective bacterial lysis, but does not exclude the subsequent emergence of phage-resistant variants or resistant subpopulations (Chan et al., 2016; Abedon, 2026), which may repopulate the culture once phage predation pressure is reduced or adsorption becomes limiting (Abedon, 2025), a phenomenon well documented in phage-bacteria co-evolution studies (Lenski and Levin, 1985; Koskella and Brockhurst, 2014).

Conversely, strains that maintained high OD_600_ values throughout the assay cannot be conclusively classified as non-responders without parallel viable counts (Abedon, 2025) since apparent growth could reflect the persistence of a resistant minority that has evaded phage action rather than complete cocktail failure (Chan et al., 2016). These observations underscore the necessity of complementing OD-based kinetics with colony-forming unit (CFU) enumeration to distinguish true bactericidal activity from transient suppression followed by regrowth (Abedon, 2026), and to accurately determine whether apparent lysis reflects a sterilising effect or merely a delay in growth dynamics (Abedon, 2025).

The lack of effective control of strain T102, despite the presence of individually active phages, may reflect mechanisms such as receptor masking, resistant subpopulations, reduced adsorption efficiency or antagonistic interactions under multi-phage conditions. Conversely, the successful lysis of strains T133 and T196, despite limited individual activity, suggests the presence of synergistic effects, potentially involving complementary receptor targeting or enhanced infection dynamics under multi-phage conditions (Hyman and Abedon, 2010; Bertozzi Silva et al., 2016; de Jonge et al., 2019; Wright et al., 2021). In this context, integrating quantitative infectivity data with computational optimization represents a key step toward more predictive cocktail development, particularly because phage combinations may display complementary, neutral or antagonistic interactions depending on the bacterial host and cocktail composition (Forti et al., 2018; Yang Y. et al., 2020; Molina et al., 2022, 2025; Koncz et al., 2024). Future validation studies should therefore incorporate parallel CFU monitoring alongside OD_600_ kinetics to provide a more complete picture of cocktail bactericidal dynamics and to reliably assess long-term suppression efficacy.

Finally, environmental stability assays showed that the selected CKT6 phages-maintained infectivity across a broad range of pH and temperature conditions compatible with many poultry-production settings, supporting their practical potential. However, their sensitivity to extreme acidic conditions indicates that formulation or encapsulation strategies may be required for application routes involving low-pH exposure, such as oral delivery (Malik et al., 2017; Richards and Malik, 2021).

From an applied perspective, CKT6 should therefore be considered a broadly active baseline formulation that could be refined according to circulating *E. faecalis* populations, production type and farm-level epidemiology. This adaptive approach may be particularly important given the strain-level variability observed in this study and the likelihood that no universal phage cocktail will provide sustained efficacy across all poultry-production scenarios. Further validation under biofilm-associated, formulation-relevant and field-like conditions will be essential before considering practical application in poultry production systems. Further studies should extend the evaluation of the selected phages under more complex and biologically relevant conditions. In particular, their activity against biofilm-associated *E. faecalis*, their interaction with antibiotics in phage-antibiotic combination approaches, and their performance in matrices that better mimic poultry production environments should be investigated. In addition, the emergence of phage-resistant variants and its impact on long-term cocktail efficacy will need to be assessed to refine cocktail composition and improve robustness. These aspects will be essential before considering field-oriented applications.

In conclusion, this study shows that phage cocktail design can evolve from empirical selection toward data-driven and mechanistically informed strategies. By integrating field-derived phage diversity, quantitative infectivity metrics, genomic characterization and computational optimization, we identified a broadly active phage cocktail targeting MDR *E. faecalis* from poultry production. Although further validation under field-relevant conditions is required, our findings highlight the value of functional complementarity and adaptive cocktail design for advancing phage-based approaches at the animal-human interface.

## 5 Conclusion

This study demonstrates that integrating large-scale EOP-based phage-host interaction profiling with computational optimization provides a framework for the rational and reproducible design of broadly active phage cocktails against MDR *Enterococcus faecalis* from poultry production. The 38-phage collection achieved 94.12% cumulative strain coverage, and the selected six-phage cocktail CKT6 suppressed 92.16% of clinical isolates at MOI 1, with efficacy not fully predictable from individual phage activity. The genomic safety profile, environmental stability, and taxonomic diversity of CKT6 components further support their potential as phage-based biocontrol candidates. Collectively, these findings advance phage cocktail design from empirical selection toward data-driven strategies, and position poultry environments as relevant reservoirs for sourcing specialized phages of One Health significance. Further validation under biofilm-associated and field-relevant conditions remains necessary before practical deployment.

## Data Availability

The datasets presented in this study can be found in the NCBI (https://www.ncbi.nlm.nih.gov) under accession numbers PZ295607.1, PZ295608.1, PZ295609.1, PZ295610.1, PZ400691.1 and PZ295606.1.
